# Rehabilitation assistive technology transfer in the AI era: network dynamics and public health impacts in the Yangtze River Delta, China

**DOI:** 10.3389/fpubh.2026.1752396

**Published:** 2026-03-30

**Authors:** Feng Hu, Huijie Yang, Xiaolong Zhou, Shuang Zhao, Liping Qiu, Shaobin Wei, Xiaoping Wang, Jiahan Hu, Yufeng Chen, Hao Hu, Haiyan Zhou

**Affiliations:** 1 Institute of International Business & Economics Innovation and Governance, Shanghai University of International Business and Economics, Shanghai, China; 2 International Business School, Shanghai University of International Business and Economics, Shanghai, China; 3 School of Law, Shanghai University of International Business and Economics, Shanghai, China; 4 College of Business Administration, Ningbo University of Finance and Economics, Ningbo, China; 5 CEEC Economic and Trade Cooperation Institute, Ningbo University, Ningbo, China; 6 College of Engineering, University of Perpetual Help System Laguna, City of Biñan, Laguna, Philippines; 7 School of Economics and Management, Zhejiang Normal University, Jinhua, China; 8 School of Economics, Shanghai University, Shanghai, China; 9 Graduate School, Nueva Ecija University of Science and Technology, Cabanatuan, Philippines

**Keywords:** artificial intelligence, geographical detector, public health, rehabilitation assistive devices, technology transfer

## Abstract

Against the backdrop of rapid global advances in artificial intelligence (AI), growing concerns surrounding public health and stressful environmental conditions, this study examines the Yangtze River Delta (YRD) of China by using social network analysis, a geographical detector model, and a coupling coordination model to investigate the spatiotemporal evolution and factors associated with rehabilitation assistive technology transfer networks before and after the COVID-19 pandemic (2016–2023). The results reveal that the YRD’s technology transfer network has continued to expand, transforming from a “single-center” structure dominated by Shanghai to a “multicenter” network jointly anchored by Shanghai, Hangzhou, and Suzhou. The results of the coupling coordination analysis indicate that the synergistic relationship between public health systems and technology transfer networks improved during the pandemic. In contrast, the synergistic potential of AI technology innovation has yet to be fully unleashed. The geographical detector results show that economic development, technological innovation, and financial development are the core drivers of technology transfer. Moreover, interactions between public health or AI inventive capacity and other determinants exhibit pronounced “bifactor enhancement” effects. This study offers a new perspective on the sustainable development of the rehabilitation assistive devices industry within the context of AI integration and environmental pressures.

## 1 Introduction

With China’s rapid population aging and accelerated technological advancement, the use of rehabilitation assistive devices, essential tools for improving the quality of life of older adults and people with disabilities, has become a critical topic in the public health domain. Since 2016, the Chinese government has introduced a series of supportive policies, including the *Opinions on Accelerating the Development of the Rehabilitation Assistive Device Industry* and the *Multiple Measures to Promote High-Quality Development of Inclusive Services for Older Adults*, creating favorable conditions for AI application and development in the assistive device industry. The advancement of society and economic growth are inextricably linked to the collective improvement of living conditions for these vulnerable groups. Moreover, how industrial integration can help the rehabilitation assistive devices industry adapt to contemporary pressures, such as climate change and environmental stress, is rarely discussed.

By the end of 2024, China had 310 million people aged 60 and above, accounting for 22% of the population.^1^ Moreover, the number of people with disabilities reached 86 million by the end of 2023, exceeding 6%.^2^ The enormous demand for assistive devices generated by older adults and people with disabilities has elevated the development of the assistive device industry, making it a major public welfare priority. In particular, given the rapid increase in AI and the *Healthy China* strategy, the research, development, and application of intelligent rehabilitation devices have become prominent industry trends.

Technology transfer is not only fundamental for transforming technological achievements into productive forces but also essential for reducing regional disparities in scientific and technological resources and lowering R&D costs. As a key mechanism for improving urban innovation capacity, technology transfer has attracted widespread scholarly and policy attention (1–7). Studies on technology transfer networks often rely on patent transactions or assignments, constructing patent-based technology transfer networks at the micro (universities and firms), meso (industries such as smartphones and ICT), and macro levels (cities or countries). Using social network analysis, scholars have examined structural features, including small-world characteristics, network density, and node attributes (3, 5, 8–26). Research on influencing factors focuses primarily on multidimensional proximities, geographical, social, and technological, as well as knowledge bases and absorptive capacity (27–38).

With respect to the measurement of public health, the academic community has gradually expanded the concept from a narrow focus on physical indicators, such as mortality and life expectancy, to a multidimensional system encompassing physiological, psychological, social, environmental, healthcare service, and socioeconomic conditions. Scholars such as Ye et al. and Zhao et al. have adopted multidimensional approaches to construct urban public health evaluation systems (39–44). For measuring AI inventive capacity, researchers typically use robot installation density, AI patents, fixed asset investment in the software industry, or AI-related publications, or they construct composite indices incorporating hardware, research capacity, and data resources (45–52).

The Yangtze River Delta (YRD) is among China’s most economically dynamic regions. It has led the nation in terms of innovation and technology transfer in the rehabilitation assistive device industry. According to the Incopat global patent database, since 2016, the YRD has accounted for more than 20% of national patent applications in this field, with its share surpassing 30% in 2021 and 2022. Numerous scholars have attempted to explore the innovation efficiency of the YRD using diverse research methods. Particularly given the region’s high population density and advanced industrial development, its green innovation efficiency has received special attention. For instance, Guijian et al. investigated the nonlinear driving factors of green innovation in the Yangtze River Economic Belt and found that industrial pollution exerts a threshold effect on innovation development, that moderate levels of pollution can boost green innovation, whereas excessive pollution hinders the innovation process (53). This study also confirms that technological diffusion and inter-city coordinated development do not advance in tandem with capacity improvement; instead, their ultimate outcomes depend on institutional and structural conditions, including the heterogeneity of regional development capacities and the interplay between environmental pressures and technological progress. Achieving inter-city coordinated development requires the support of targeted institutional mechanisms, rather than relying solely on market forces or the development capacity of individual cities. For the rehabilitation assistive device industry, this implies that the construction of technology transfer networks must facilitate knowledge sharing, align policy objectives across administrative regions, and address the heterogeneity of institutional capacities among cities at different developmental stages. These strategies have been explored and validated in numerous studies, boasting both scientific rigor and practical effectiveness (54, 55).

The YRD, centered on Shanghai, hosts a large number of universities, research institutes, and high-tech enterprises, providing essential support for the transformation and application of AI technologies in the rehabilitation assistive device sector. The integration of AI and assistive rehabilitation technologies may also contribute to advancing a low-carbon economy, a circular economy, and a health economy. AI-driven devices can optimize energy efficiency during use, extend product lifespans through predictive maintenance, and promote remote rehabilitation models to reduce transportation demands. Furthermore, AI can optimize design and manufacturing processes, minimize material waste, and encourage sustainable resource utilization. Therefore, the transfer and adoption of such intelligent technologies represent not only healthcare innovation but also an effective pathway to reduce the carbon footprint in the medical field and foster more sustainable consumption and production patterns within the assistive technology industry.

However, how to foster closer synergy between AI technology and the rehabilitation assistive device industry, and explore the translation pathways of relevant research outcomes into industrial applications to respond to the growing demand for public health services, remains an important practical issue that urgently needs to be clarified. Meanwhile, the COVID-19 pandemic has caused significant disruptions to global public health systems. Against this backdrop, whether the rehabilitation assistive device technology transfer network in the YRD has exhibited structural changes before and after the pandemic, and how these changes are linked to regional public health capacity and technological development levels, awaits further systematic analysis. Therefore, this study focuses on the YRD and examines the spatiotemporal evolution and determinants of the technology transfer network before and after the pandemic (2016–2023) by using social network analysis, a geographical detector model, and a coupling coordination model. The findings aim to inform policy design in the post-pandemic era, promote more efficient allocation of innovation resources, and enhance public health outcomes.

## 2 Research methods and data sources

### 2.1 Research methods

#### 2.1.1 Social network analysis

The technology transfer network of rehabilitation assistive devices in the YRD, constructed via social network analysis, is directed and weighted. Nodes represent prefecture-level cities in the YRD. Directed edges point from the assignor’s city (technology outflow city) to the assignee’s city (technology inflow city), indicating the direction of technology flow. Edge weights denote the total number of patent transfer events between two cities in the research period. Using Gephi software, the fundamental network attributes, such as network density and average degree, and node-level features, including weighted in-degree and out-degree, betweenness centrality, and closeness centrality, were measured (56–60).

#### 2.1.2 Geographical detector

The factor detector module was used to examine the relationships between the weighted in-degree/out-degree of technology transfer networks and determinants such as economic development and scientific innovation. The interaction detector was further employed to examine the interaction effects between public health, AI inventive capacity, and other selected factors in relation to the technology transfer network (61–64).

#### 2.1.3 Coupling coordination model

The model was used to calculate the degree of coupling coordination between nodes weighted in−/out-degree, and urban public health and AI technology innovation levels in the YRD (65–67).

### 2.2 Data sources

#### 2.2.1 AI technology innovation level

Following the relevant literature (39–43), 78,506 AI-related patents in the YRD were retrieved from the Incopat database, with 67,100 patents from 2016 to 2023 used for analysis.

#### 2.2.2 Public health level

Drawing on the research perspectives of relevant scholars (44–52), this paper adopts the entropy value method to conduct a multi-dimensional comprehensive evaluation from three dimensions, with the number of hospital beds per 10,000 people and the number of physicians per 10,000 people as the foundation of public health, industrial sulfur dioxide emissions as the environmental exposure risk, and Baidu Index for depression, anxiety and depression as well as the incidence of infectious diseases as the public health performance. Given that the data on the incidence of infectious diseases are only available at the provincial level, and this indicator is positively correlated with the urban population size while negatively correlated with the urban level of science and technology (68), the urban incidence of infectious diseases in the YRD is finally obtained by weighting the above two indicators based on their proportions at the provincial level.

#### 2.2.3 Transfer networks of rehabilitation assistive device technology

First, the national economic industry code (3586) for the rehabilitation assistive device manufacturing industry was determined in accordance with the *Strategic Emerging Industries Classification* issued by the National Bureau of Statistics. China’s patents related to rehabilitation assistive device technologies were retrieved from the Incopat patent database, and a total of 10,718 patents with transfer records in their legal events were screened and downloaded. From the “legal status” of the patent data, the patent transfer time, and the addresses before and after the change were collated, overseas addresses were excluded, and the corresponding provinces and cities of the remaining addresses were matched based on the address information. Subsequently, a rigorous process involving extensive manual verification, cross-checking, and random sampling was conducted to ultimately screen out the patent transfers associated with the YRD. In the first stage, by verifying the address information of both patent assignors and assignees, only those patents where both parties were located in cities of the YRD were retained. In the second stage, cross-verification was performed on the results of the initial screening. Two researchers mutually reviewed the patents retained by each other, and all discrepancies were documented, including inconsistent judgments on whether a city belonged to the YRD and whether the address information was sufficient to support geolocation. In the third stage, to ensure data quality, a third researcher conducted an independent verification on a 10% random sample of the retained patents, with a 100% agreement rate achieved in this sampling verification. Following the above stringent verification procedures, a total of 558 valid patents related to the YRD for the period 2016–2023 were finally identified. Third, technological transfer events were defined according to the urban changes before and after patent transfers: a transfer from City A (before the change) to City B (after the change) was recorded as one technological transfer event. On this basis, the technological transfer matrices of rehabilitation assistive devices in the YRD were constructed for two periods, 2016–2019 and 2020–2023, respectively.

## 3 Current status of AI inventive capacity and public health in the YRD

### 3.1 Temporal evolution of AI inventive capacity and public health

**Figure 1:**
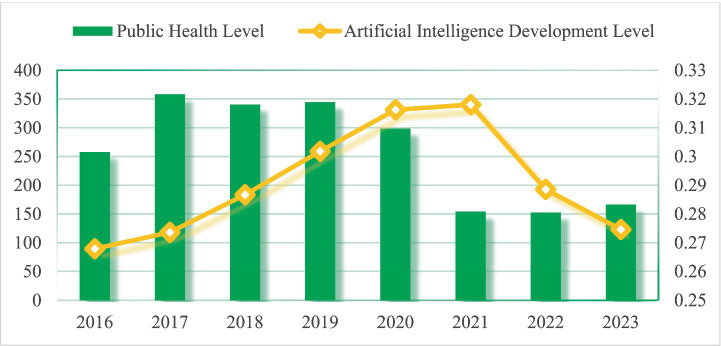
Time-series evolution of AI inventive capacity and public health levels in YRD cities.

As shown in Figure 1, during the 2016–2019 period, the average public health level in YRD cities remained relatively stable at approximately 0.32. In the 2020–2023 period, however, a decline in public health levels was observed across most cities, coinciding with the global COVID-19 pandemic. In contrast, AI inventive capacity exhibited a different trajectory, which increased substantially between 2016 and 2021, particularly during 2020–2021 when the pandemic was most acute, before declining sharply in the subsequent post-pandemic period. These temporal patterns are consistent with broader trends documented in the literature. According to a global survey by the IBM Institute for Business Value, the pandemic period witnessed accelerated adoption of AI applications, such as intelligent temperature screening and digital mobility tracking, moving AI from experimentation toward wider implementation.^3^ Conversely, a CB Insights report indicates that by 2023, AI financing in China had declined by approximately 70% compared to the peak period, suggesting a gradual return to more rational market expectations.^4^ While these co-occurring trends are suggestive, the extent to which the observed changes in the YRD can be attributed specifically to the pandemic, as opposed to concurrent policy initiatives, technological maturation, or other factors, remains an important question for future research.

### 3.2 Spatial evolution of AI inventive capacity and public health levels in YRD cities

**Figure 2:**
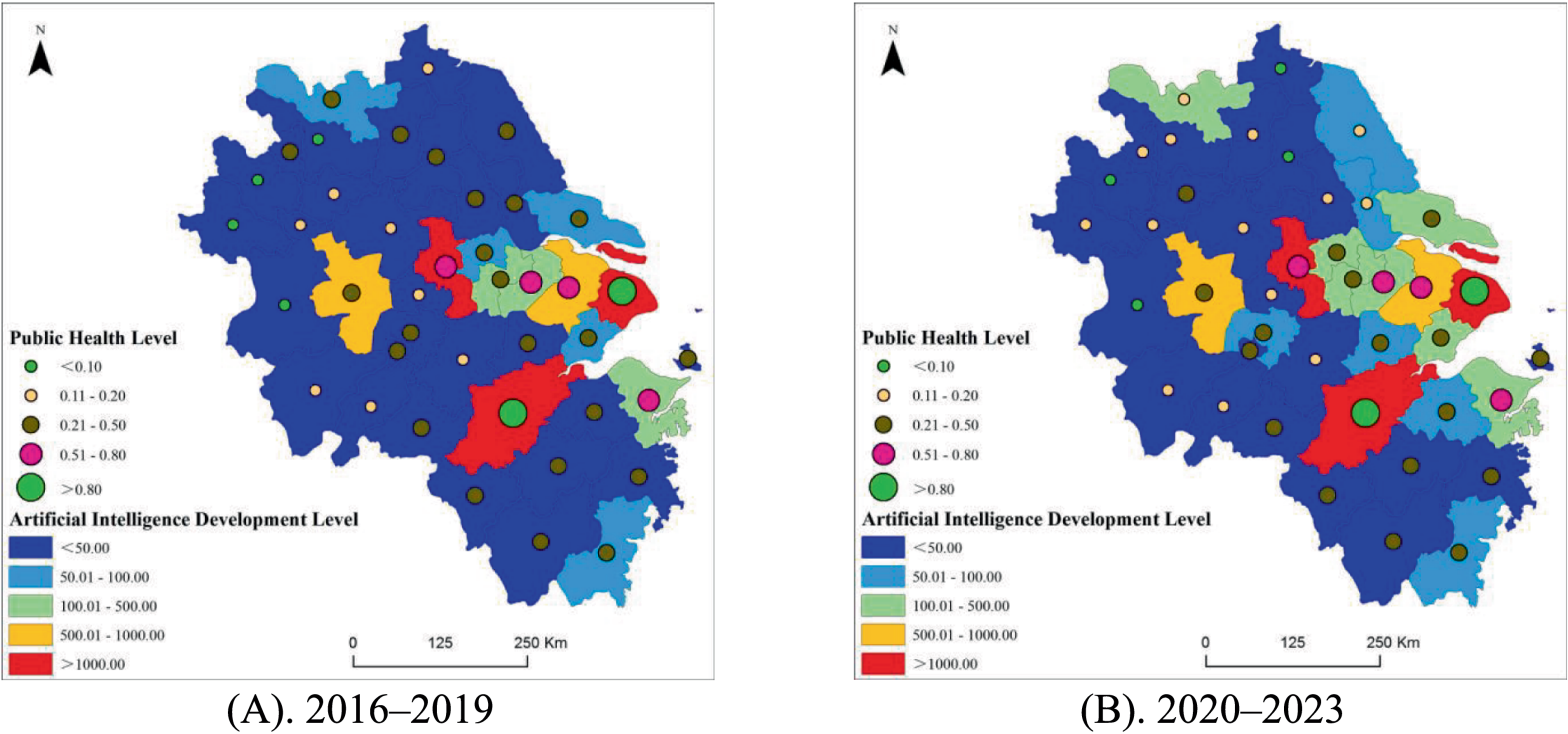
Spatial pattern evolution of AI inventive capacity and public health levels in YRD cities. **(A)** 2016–2019; **(B)** 2020–2023.

As shown in Figure 2, most cities did not see improvements in public health levels during the COVID-19 pandemic. Core cities such as Shanghai, Hangzhou, and Nanjing consistently maintained the highest public health levels, demonstrating strong resilience in their public health systems. Cities such as Ningbo and Jiaxing experienced modest but steady increases and maintained relatively high public health levels. However, more than half of the cities, including Changzhou, Hefei, Suzhou, Wuxi, Wenzhou, Huai’an, and Lianyungang, experienced significant declines during the pandemic, with many falling to middle or low levels of public health. Overall, the results reveal considerable heterogeneity in public health capacity across cities, with core metropolitan areas maintaining relatively stronger positions than smaller cities.

In contrast, the level of AI inventive capacity increased significantly in most cities. As regional leaders, Shanghai and Hangzhou exceeded the AI inventive capacity index of 2000, while Nanjing approached 1,600, consistently occupying the top tier of AI inventive capacity. Both Hefei and Suzhou advanced from the middle to upper-middle level of AI inventive capacity. Many cities achieved substantial upward mobility across levels. For example, Jiaxing’s AI index rose from 54.5 (low level) to 135.25 (medium level), and Nantong’s increased from 55 to 154.25, indicating that these cities entered a new stage of AI industrial development. The AI inventive capacity levels of even smaller third- and fourth-tier cities with weaker foundations, such as Bengbu, Chizhou, and Suqian, increased several-fold (Table 1).

**Table 1:** Attributes of the technology transfer network of rehabilitation assistive devices in the YRD cities.

Network attributes	2016–2019	2020–2023
Nodes	23	32
Edges	59	124
Average degree	2.565	3.875
Average weighted degree	14.522	16.469

## 4 Yangtze River Delta rehabilitation assistive technology transfer network

### 4.1 Overall network attributes

A comparative analysis of the technology transfer network of rehabilitation assistive devices in the YRD before and after the COVID-19 pandemic revealed significant development and optimization in both network scale and structure. The network expanded steadily, with the number of participating cities increasing from 23 to 32. Between 2020 and 2023, Huaibei and Suzhou withdrew from the network, while Bozhou, Chizhou, Huainan, Huangshan, Lu’an, Ma’anshan, and Yangzhou never participated. Except for Yangzhou in Jiangsu Province, the remaining cities are located in Anhui Province, accounting for half of Anhui Province’s total cities. The number of technology transfer connections increased sharply from 59 to 124, indicating closer interactions between cities (the average degree rose from 2.565 to 3.875). Moreover, the network structure has become increasingly mature: not only has the intensity of technology transfer increased (average weighted degree from 14.522 to 16.469), but the overall network has also become more cohesive. These changes collectively demonstrate the gradual maturity and integration of the rehabilitation assistive technology transfer network in the YRD. During the 2020–2023 period, the technology transfer network in this region did not exhibit disruptions or negative impacts. During the pandemic, both national and local governments promoted the development of smart healthcare and rehabilitation assistive devices through policies such as the “14th Five-Year National Health Plan” and the “‘Robotics+’ Application Action Implementation Plan,” providing support through fiscal subsidies and government procurement. Additionally, this period coincides with growing market interest and economic potential in smart care technologies and rehabilitation robots (52), which may create additional momentum for technology transfer in the field of rehabilitation assistive devices.

### 4.2 Analysis of individual network position

**Figure 3:**
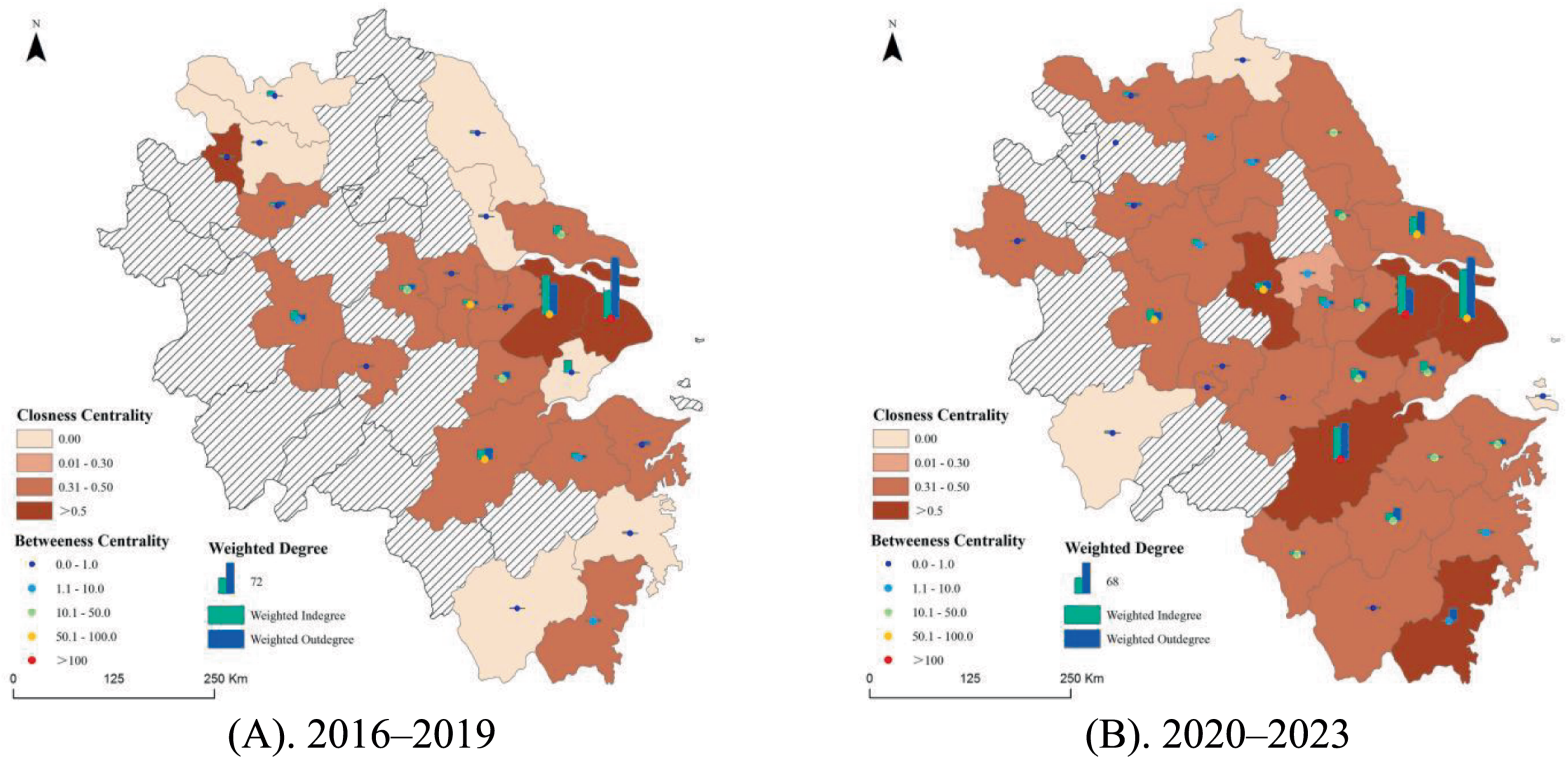
Individual network position in the rehabilitation assistive technology transfer network of the YRD. **(A)** 2016–2019; **(B)** 2020–2023.

As shown in Figure 3, in terms of weighted in-degree and out-degree, during Phase 1, Shanghai and Suzhou served as the core of the rehabilitation assistive technology transfer network in the YRD, demonstrating strong capabilities in both technology absorption and diffusion. Cities such as Hangzhou, Hefei, and Nanjing also exhibited relatively high in-degree and out-degree, whereas cities such as Jiaxing (out-degree = 0) and Ningbo (in-degree = 0) demonstrated unidirectional technology flow. In Phase 2, with the expansion of the technology transfer network, more cities participated: Shanghai and Suzhou remained central, but Hangzhou’s in-degree and out-degree increased significantly, becoming another key node. The in-degree and out-degree of Hefei, Nanjing, and Nantong also increased noticeably, indicating more active and balanced technology transfer activities.

With respect to closeness centrality, in Phase 1, only Shanghai and Suzhou reached the highest levels, whereas most cities, including Hangzhou, Nanjing, and Hefei, were at moderate levels. Cities such as Jiaxing and Lishui had a closeness of 0 and were located on the network’s periphery. In Phase 2, the number of cities with the highest closeness increased, and the influence of core cities expanded. The group of moderately central cities expanded, bringing more cities into the main backbone of technology transfer, while the number of peripheral cities decreased to only a few, namely, Anqing, Lianyungang, and Zhoushan.

In terms of betweenness centrality, during Phase 1, Shanghai acted as the core bridge of the rehabilitation assistive technology transfer network, while Hangzhou and Suzhou were secondary core intermediaries. Cities such as Nanjing, Huzhou, and Nantong played relatively crucial connecting roles and were key intermediary cities, whereas most other cities had low or zero betweenness. In Phase 2, the number of core bridging cities increased to include Hangzhou and Suzhou, with Shanghai shifting to a secondary intermediary role. Hefei and Nanjing also entered the secondary intermediary group. The number of important intermediary cities, such as Nantong, Jiaxing, and Jinhua, increased, although many cities (e.g., Anqing and Wuhu) still had a betweenness centrality of 0.

Overall, the technology transfer network of rehabilitation assistive devices in the YRD exhibited greater resilience during the 2020–2023 period. Shanghai’s control capacity declined, while Suzhou and Hangzhou gradually emerged as major collaborative hubs within the network. This trend is also supported by real-world examples; for instance, a report by *People’s Daily* highlighted that Zhikangjia Company in Shanghai’s Putuo District employed its portable upper-limb rehabilitation robot technology in medical and residential care facilities for the older population in Nantong, Jiangsu, through industry connections.^5^ This case not only demonstrates Shanghai’s capacity to diffuse rehabilitation assistive device technologies but also reflects the rise of cities, such as Nantong, as hubs for technology reception and application.

### 4.3 Spatial pattern of the network

In Phase 1, the technology transfer network of rehabilitation assistive devices in the YRD exhibited a high degree of centralization. Shanghai was the undisputed core, exporting the greatest number of high-intensity technology transfers in diverse directions. Notably, the bidirectional high-intensity technology transfers between Shanghai and Suzhou, reaching 85 and 57, respectively, formed the “main axis” of the network. The intensity of technology transfers among other cities was generally much lower.

In Phase 2, the network’s complexity and balance improved significantly. First, although Shanghai’s influence remained strong, the bidirectional high-intensity connection between Shanghai and Hangzhou replaced the former Shanghai–Suzhou main axis, becoming the strongest technology transfer channel in the network. This shift indicates the rapid rise of Hangzhou as a key hub. Second, Nantong’s importance as a technology transfer source became more prominent, with particularly high-intensity links directed toward Shanghai. Additionally, cities such as Jinhua and Wenzhou emerged as new active sources of technology diffusion, establishing medium- to high-intensity connections with core nodes such as Suzhou and Hangzhou.

**Figure 4:**
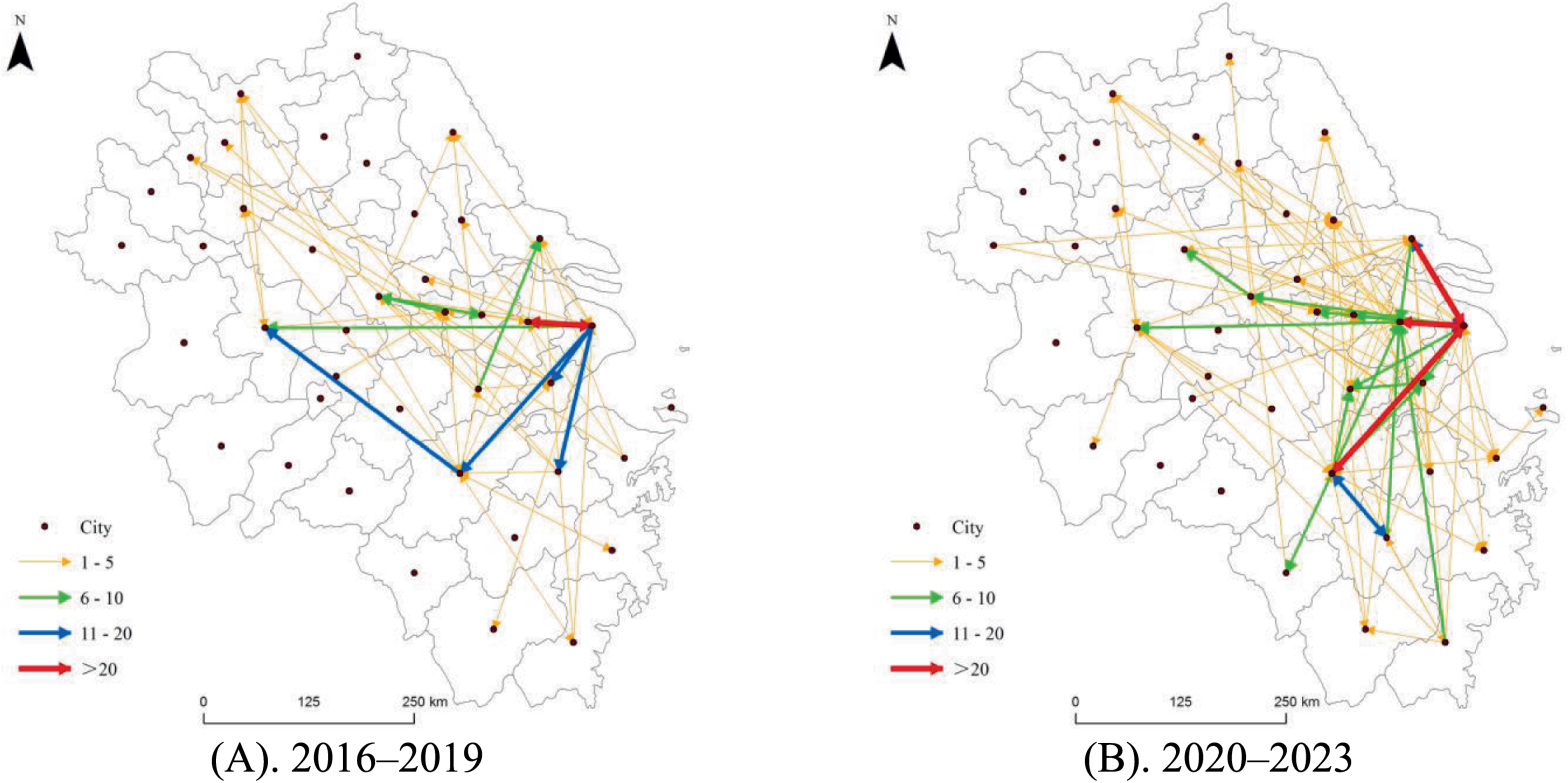
Spatial pattern of rehabilitation assistive technology transfer networks in the YRD. **(A)** 2016–2019; **(B)** 2020–2023.

In summary, the spatial pattern of the rehabilitation assistive technology transfer network in the YRD transitioned after the pandemic from a single-center radiation model that depended on Shanghai to a multicenter network structure with Shanghai, Hangzhou, and Suzhou as key hubs (Figure 4).

## 5 Coupling coordination analysis

### 5.1 Temporal variation characteristics

**Figure 5:**
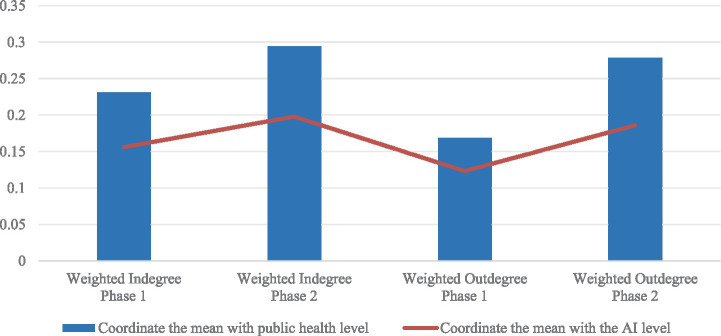
Coupled coordination mean value in the YRD.

From Phase 1 to Phase 2, the degree of coupling coordination between urban public health levels, AI inventive capacity, and the weighted in-degree and out-degree of the rehabilitation assistive technology transfer network in the YRD markedly improved. Specifically, the mean degree of coupling coordination between public health levels and the weighted in-degree and out-degree increased from 0.231 and 0.168 to 0.294 and 0.278, respectively. Similarly, the mean degree of coupling coordination between AI inventive capacity levels and the weighted in-degree and out-degree increased from 0.157 and 0.123 to 0.198 and 0.186, respectively. These results indicate that during the 2020–2023 period, the overall synergistic relationship between public health, AI, and the transfer of rehabilitation assistive device technology in the YRD was stronger than in the previous period, with the improvement in public health coupling coordination being particularly pronounced (Figure 5).

### 5.2 Spatial variation characteristics

Using grading standards referenced from relevant studies (69), a more in-depth analysis of the coupling coordination of cities in the YRD revealed the following. In Phase 1, only a few cities, such as Suzhou and Shanghai, reached high-level coordination in terms of weighted in-degree. In Phase 2, cities such as Hangzhou and Suzhou achieved high-level coordination across multiple indicators, and the number of cities with basic coordination increased significantly. In particular, cities such as Jinhua, Nanjing, and Nantong experienced notable improvements in weighted out-degree, reflecting a stronger synergistic relationship between the public health system and the technology transfer network in the 2020–2023 period. Nevertheless, some cities, including Wenzhou, Yancheng, and Zhenjiang, experienced a declining coordination level or persistent severe disorder.

In contrast, the coupling coordination between AI inventive capacity levels and the technology transfer network was weaker overall. Although a few cities (e.g., Hangzhou, Hefei, Nanjing) maintained at least basic coordination in some indicators during Phase 2, the number of cities experiencing severe disorder remained much higher than that in the public health system. In particular, for the weighted out-degree, most cities were still in a state of severe disorder. This indicates that a significant conversion gap exists between AI knowledge production and technology transfer in most cities. This gap likely stems from several interconnected barriers: first, the interdisciplinary challenges encountered when integrating AI technology with clinical rehabilitation needs; second, the absence of standards and efficacy validation mechanisms for smart rehabilitation assistive devices; and third, the inadequacy of supporting institutional systems for technology transfer in peripheral and medium-sized cities. The study’s findings also underscore that unlocking the potential of AI technology within rehabilitation assistive device technology transfer networks hinges on bridging this conversion gap, not merely increasing patent output.

Overall, during the pandemic, the coupling coordination between the public health system and the technology transfer network improved notably, whereas the synergistic effectiveness of AI has yet to be fully realized.

## 6 Factors influencing the rehabilitation assistive technology transfer network in the YRD

### 6.1 Variable selection

Drawing on the theory of technology transfer and innovation geography, as well as the perspectives of previous scholars (27–38), this study posits that the transfer of rehabilitation assistive device technologies not only follows general technology transfer patterns but is also strongly related to urban public health and AI inventive capacity. Therefore, following the principles of comprehensiveness and data availability, the weighted out-degree, weighted in-degree, betweenness centrality, and closeness centrality of the rehabilitation assistive technology transfer network were selected as the dependent variables. Independent variables were chosen to reflect economic development (GDP), technological innovation (number of patent grants), openness to external trade (total imports and exports), residents’ consumption level (total retail sales of consumer goods), financial development (year-end balance of RMB loans in financial institutions), infrastructure level (telecommunications and postal service revenue), public health level, and AI inventive capacity level. Before the analysis was conducted with the geographical detector method, all the variables were classified into five levels using the natural breaks method in ArcGIS. Data on influencing factors were obtained from the 2024 *China City Statistical Yearbook*.

### 6.2 Analysis results

Geographical detector analysis revealed that all influencing factors exhibited significant explanatory power (mostly significant at the 1% level), although the strength of their effects varied notably.

First, regarding the network’s control and radiation capabilities (weighted in-degree and out-degree), economic development and technological innovation levels showed an explanatory power exceeding 0.82 for both weighted in-degree and out-degree, ranking first and second. These findings indicate that economically advanced and technologically leading cities strongly attract and disseminate rehabilitation assistive device technologies. Additionally, financial development and infrastructure levels also exhibited considerable explanatory power for weighted out-degree, suggesting that strong financial support and well-developed infrastructure are key enablers of outward technology transfer.

Second, in terms of key control over the network structure (betweenness centrality), financial development emerged as the most prominent factor, with a Q value of 0.814, ranking highest among all factors. This finding indicates that cities with a high level of financial development serve as indispensable “intermediary hubs” in the rehabilitation assistive technology transfer network, effectively connecting different nodes and facilitating smooth and integrated technology transfers. Economic development and technological innovation levels followed closely, with high explanatory power.

Finally, regarding network accessibility and independence (closeness centrality), financial development again demonstrated strong explanatory power. This implies that financially developed cities are less likely to be “isolated” from other nodes and can establish connections with other members of the network through shorter paths, positioning them at the network’s core. Residents’ consumption levels also showed strong explanatory power for closeness centrality, suggesting that the consumption capacity of urban residents is a crucial factor in attracting rehabilitation assistive device resources and enhancing a city’s centrality within the network.

It is noteworthy that AI invention and innovation capabilities demonstrate strong explanatory power for all centrality metrics (with Q-values exceeding 0.77 for weighted in-degree, weighted out-degree, and betweenness centrality). However, as shown by the coupling coordination analysis results in Table 2, significant regional disparities exist in the alignment between AI invention and innovation capabilities and actual technology transfer flows across cities in the YRD. Core hub cities exhibit higher alignment between the two, while most other cities show relatively low alignment. This characteristic indicates that AI knowledge production capacity is a necessary but not sufficient condition for technology transfer. Without supporting technology conversion infrastructure and institutional reception capabilities, upstream AI innovation achievements struggle to translate into downstream technology diffusion effectiveness. This conversion gap is particularly pronounced in peripheral and medium-sized cities (Table 3).

**Table 2:** Urban coupling coordination levels in the YRD.

Coupling coordination type	Coordinated with public health levels				Coordinated coupling with AI levels			
	Weighted in-degree phase 1	Weighted in-degree phase 2	Weighted out-degree phase 1	Weighted out-degree phase 2	Weighted in-degree phase 1	Weighted in-degree phase 2	Weighted out-degree phase 1	Weighted out-degree phase 2
Senior Coordination 0.8 < D ≤ 1	Suzhou	Hangzhou, Suzhou		Hangzhou		Hangzhou		Hangzhou
	Shanghai							
Basic Coordination 0.5 < D ≤ 0.8	Hangzhou, Hefei, Jiaxing, Nantong	Changzhou, Hefei, Huzhou, Jiaxing, Jinhua, Nanjing, Nantong, Wuxi	Hangzhou, Suzhou	Jinhua, Nanjing, Nantong, Suzhou, Wenzhou	Hangzhou, Hefei, Suzhou	Hefei, Suzhou, Nanjing	Hangzhou, Suzhou	Suzhou, Nanjing
Basic Discoordination 0.3 < D ≤ 0.5	Bengbu, Changzhou, Huzhou, Huaibei, Nanjing, Shaoxing, Taizhou (Jiangsu), Wuxi, Xuzhou, Yancheng, Zhenjiang	Bengbu, Chuzhou, Ningbo, Quzhou, Shaoxing, Taizhou (Zhejiang), Taizhou (Jiangsu) Xuzhou	Bengbu, Changzhou, Hefei, Huzhou, Nanjing, Ningbo, Shaoxing, Wenzhou, Wuxi	Bengbu, Changzhou, Hefei, Huzhou, Jiaxing, Ningbo, Quzhou, Wuxi	Nanjing, Jiaxing	Changzhou, Wuxi, Nantong, Jiaxing	Hefei, Nanjing	Hefei, Wuxi, Nantong
Senior Discoordination 0 < D ≤ 0.3	Chuzhou, Jinhua, Ningbo, Quzhou, Wenzhou	Huai’an, Wenzhou, Yancheng, Zhenjiang	Chuzhou, Huaibei, Jiaxing, Jinhua, Nantong, Quzhou, Taizhou (Zhejiang), Taizhou (Jiangsu), Xuzhou, Yancheng, Zhenjiang	Chuzhou, Huaibei, Shaoxing, Taizhou (Zhejiang), Taizhou (Jiangsu), Xuzhou, Yancheng, Zhenjiang	Changzhou, Nantong, Wuxi		Changzhou, Jiaxing, Nantong, Wuxi	Changzhou, Jiaxing
	Anqing, Bozhou, Chizhou, Fuyang, Huai’an, Huainan, Huangshan, Lishui, Lianyungang, Lu′an, Ma’anshan, Tongling, Wuhu, Suqian, Suzhou, Xuancheng, Yangzhou, Zhoushan				Anqing, Bengbu, Bozhou, Chizhou, Chuzhou, Fuyang, Huzhou, Huai’an, Huaibei, Huainan, Huangshan, Jinhua, Lishui, Lianyungang, Lu′an, Ma’anshan, Ningbo, Quzhou, Shaoxing, Taizhou (Zhejiang), Taizhou (Jiangsu), Tongling, Wenzhou, Wuhu, Suqian, Suzhou, Xuzhou, Xuancheng, Yancheng, Yangzhou, Zhenjiang, Zhoushan			

**Table 3:** Factor detection analysis results.

Influencing factors	Indicators	Weighted in-degree		Weighted out-degree		Betweenness centrality		Closeness centrality	
		Q-value	*p* value	Q-value	*p* value	Q-value	*p* value	Q-value	*p* value
Level of economic development	GDP	0.821	0.000	0.860	0.000	0.837	0.000	0.646	0.003
Level of technological innovation	Number of patents granted	0.778	0.000	0.824	0.000	0.785	0.000	0.654	0.002
Level of trade openness	Value of goods imported and exported	0.685	0.007	0.753	0.000	0.672	0.006	0.537	0.065
Level of resident consumption	Total retail sales of consumer goods	0.705	0.000	0.784	0.000	0.741	0.000	0.678	0.000
Level of public health	Public health levels	0.658	0.000	0.789	0.000	0.580	0.000	0.544	0.007
Level of AI inventive capacity	AI inventive capacity level	0.778	0.000	0.779	0.000	0.784	0.000	0.612	0.007
Level of financial development	Year-end balance of RMB loans by financial institutions	0.757	0.000	0.785	0.000	0.814	0.000	0.760	0.000
Level of infrastructure	Revenue from telecommunications and postal services	0.731	0.000	0.793	0.000	0.627	0.000	0.671	0.000

Further analysis using interaction detection revealed that interactions among public health levels, AI inventive capacity levels, and any other influencing factors consistently exhibit dual-factor enhancement. In other words, the Q values of all the interaction terms exceeded those of the individual factors. This finding indicates that the rehabilitation assistive technology transfer network in the YRD is driven by a complex system of influencing factors and that the synergistic effects among these factors are far stronger than the independent effects of either public health levels or AI inventive capacity alone.

The interaction between public health levels and economic development levels was the most prominent, particularly for the weighted out-degree of technology diffusion (Q = 0.955) and the weighted in-degree of technology reception (Q = 0.895), both reaching the highest values. This may reflect the demand-driven nature of assistive technology diffusion in rehabilitation. Cities with more developed public health systems typically possess larger healthcare networks, richer rehabilitation facility resources, and stronger demand for assistive devices. When these conditions combine with higher levels of economic development, they create a favorable environment for technology adoption and diffusion. The powerful synergistic effects between the two factors enable cities to effectively attract rehabilitation assistive technology while also becoming important sources for disseminating technology outward.

The interaction of the level of AI inventive capacity with other factors also demonstrated strong driving effects. Its interaction with technological innovation levels contributed most to the increase in betweenness centrality, indicating that the “AI + Technology” innovation combination can greatly enhance a city’s hub control and bridging role within the technology transfer network of rehabilitation assistive devices. This is primarily due to technological convergence. As a fusion of general technology and rehabilitation assistive device knowledge, AI technology positions cities as indispensable knowledge intermediaries in this field. Moreover, interactions between AI inventive capacity and financial development or infrastructure levels had the most pronounced effect on closeness centrality, implying that the “AI + Finance” and “AI + Infrastructure” combinations can effectively shorten the distance between a city and other nodes in the network, enhancing its accessibility and centrality. This occurs because financial development reduces information asymmetry and transaction costs, while infrastructure construction lowers communication costs. Both mechanisms enhance connectivity efficiency by shortening network distances between cities. These enabling effects on transactions and communication help cities better embed themselves within networks and improve their own network accessibility. The strong interaction effects of the interaction of the level of AI inventive capacity with other factors stand in stark contrast to the weakly coupled coordination characteristics confirmed earlier. This contradiction underscores that unlocking the latent potential of AI technologies requires not only possessing the requisite technical capabilities but also relying on robust technology transfer infrastructure and institutional implementation capacity to bridge the gap between upstream innovation and downstream diffusion (Table 4).

**Table 4:** Results of interaction detection analysis.

Interactions		Weighted in-degree	Weighted out-degree	Betweenness centrality	Closeness centrality
Public health levels	Level of economic development	0.895	0.955	0.892	0.735
Public health levels	Level of Technological Innovation	0.835	0.904	0.821	0.716
Public health levels	Level of Trade Openness	0.851	0.903	0.808	0.694
Public health levels	Level of Resident Consumption	0.853	0.949	0.816	0.755
Public health levels	Level of Financial Development	0.817	0.888	0.849	0.774
Public health levels	Level of Infrastructure	0.848	0.904	0.839	0.751
Level of AI inventive capacity	Level of economic development	0.854	0.883	0.891	0.684
Level of AI inventive capacity	Level of Technological Innovation	0.862	0.863	0.910	0.731
Level of AI inventive capacity	Level of Trade Openness	0.906	0.888	0.893	0.711
Level of AI inventive capacity	Level of Resident Consumption	0.803	0.850	0.835	0.722
Level of AI inventive capacity	Public health levels	0.844	0.933	0.828	0.719
Level of AI inventive capacity	Level of Financial Development	0.831	0.838	0.835	0.772
Level of AI inventive capacity	Level of Infrastructure	0.883	0.888	0.905	0.774

## 7 Conclusions and policy implications

### 7.1 Conclusion

From 2016 to 2023, the rehabilitation assistive technology transfer network in the YRD exhibited significant expansion and optimization before and after the pandemic, with tighter network connections. The spatial pattern of the network transformed from a “main axis” structure dominated solely by Shanghai into a multicenter structure with Shanghai, Hangzhou, and Suzhou as key hubs, with Hangzhou’s hub status rising rapidly.

In contrast to the notable decline in public health levels during the pandemic, the rehabilitation assistive technology transfer network expanded and became more connected during the 2020–2023 period, suggesting that the demand for rehabilitation assistive device technologies remained high even under pandemic conditions.

The coupling coordination between public health levels and the technology transfer network of rehabilitation assistive devices, particularly the weighted out-degree, reflecting technology diffusion capacity, improved significantly. This finding indicates that cities with higher public health levels may provide stable markets and environments for the application and diffusion of rehabilitation assistive device technologies. In contrast, the coordination between AI inventive capacity levels and the technology transfer network was generally weaker, indicating that the conversion and synergistic application of AI technologies in this field remain immature, and AI’s immense potential to drive the industry’s transition toward a circular economy model is being stifled.

Economic development and technological innovation levels are critical determinants of cities’ capacity to absorb and diffuse rehabilitation assistive device technologies. Financial development plays a key “intermediary hub” role in the network. More importantly, the interactions between public health levels, AI inventive capacity, and all other influencing factors generate “1 + 1 > 2″ synergistic effects, highlighting that the transfer of rehabilitation assistive device technologies in the YRD is driven by a complex system of multiple interacting factors and requires comprehensive consideration when promoting green technology development.

### 7.2 Implications and recommendations

The first set of strategies prioritizes upstream innovation expansion, increasing the supply of technological inventions through R&D subsidies, patent-oriented industrial policies, and support for university-industry research collaboration. Geographical detector analysis in this study confirms that the level of technological innovation is the primary driver of both technology absorption and diffusion. This strategy is particularly applicable to core hub cities such as Shanghai, Hangzhou, and Suzhou. For these cities, sustained increased investment in upstream R&D helps consolidate their leading position while transferring new technological achievements to downstream regions. Specific actionable policy instruments include: increasing funding for industry-university-research collaborative research, implementing patent application subsidies, and building specialized innovation parks focused on rehabilitation technology.

The second set of strategies focuses on the development of technology translation and diffusion infrastructure, with the core goal of converting technological inventions into deployable rehabilitation solutions. Specific implementation measures for this strategy can include establishing cross-city technology transfer platforms, coordinating and unifying cross-regional procurement mechanisms, formulating unified technical interoperability standards, developing targeted financial products for technology translation, and promoting the practical implementation and application of rehabilitation technologies.

The third set of strategies prioritizes the development and readiness of the public health system. Coupling coordination analysis shows that during the period 2020–2023, the synergistic correlation between the level of public health development and technology diffusion improved significantly, while the enabling effect of artificial intelligence technology remained relatively limited. A well-established public health system can create stable market demand for rehabilitation technologies and drive their implementation in healthcare service delivery. In particular, the improvement of rehabilitation service infrastructure and the strengthening of professional talent training can enhance the capacity of local medical institutions to integrate new technologies into clinical practice. For peripheral cities in Anhui Province that have not yet been integrated into the technology transfer network, such as Bozhou and Chizhou, improving the construction of public health infrastructure and the supporting policy system is an important prerequisite for their effective participation in technology transfer activities.

The final set of strategies is to deepen the technological integration of artificial intelligence with rehabilitation assistive devices. According to the results of the interaction detector, the interaction between the level of AI inventive capacity and other factors exhibits a strong driving force. Policy can unlock this driving force through three synergistic pathways: First, health departments issue “demand lists” in the rehabilitation field to guide AI R&D to focus on high-value application scenarios and avoid the disconnection between technological supply and clinical practice. Second, establish “fast-track channels” for core research institutions, providing access to data collection platforms and green channels for ethical review to accelerate the translation of AI rehabilitation products from patents to tangible goods.

### 7.3 Theoretical contributions

1. This study integrates three relatively independent research fields, public health, artificial intelligence, and technology transfer networks, to construct a multidimensional, cross-disciplinary research framework. This novel framework overcomes the limitations of prior studies that focused solely on technology or a single industrial domain, enriching the application of innovation geography in health research and promoting interdisciplinary integration between innovation geography and health studies (18–22, 43, 44, 47–51).
2. By employing a geographical detector and interaction detection, this study empirically revealed the critical roles of various influencing factors. Public health and AI, in particular, synergistically drive the technology transfer network of rehabilitation assistive devices through a “dual-factor enhancement” mechanism, uncovering a multifactor interaction driving mechanism (33–38).

### 7.4 Limitations and future research

1. In this study, the measurement of public health levels relies on provincial estimates of infectious disease incidence and the Baidu Index for mental health indicators, which may limit precision and reliability and fail to fully capture city-level public health status. Similarly, measuring AI development solely by patent counts reflects technological innovation but fails to capture the actual application level of AI technologies. Future research could incorporate indicators of AI industry implementation outcomes, such as the number of approved medical AI devices, metrics related to clinical applications, and the scale of AI investment in the healthcare sector.
2. The analysis of the rehabilitation technology transfer network is based on 558 technology transfer patents, a relatively small sample. This may limit the conclusions derived from the network analysis and further indicates that China’s rehabilitation assistive technology transfer market is generally less active, characterized by uneven technology conversion. Furthermore, this study only focuses on the quantitative dimension of technology transfer (i.e., the number of patent transfers) and does not cover qualitative dimensions such as patent complexity and innovativeness. Owing to data limitations, it also fails to incorporate the subject attributes of patent holders and the circumstances involving multiple patent holders into the analysis. Future research could conduct in-depth investigations into the aforementioned aspects.
3. The study conclusions are based on the YRD, one of China’s most economically and technologically developed regions. Whether these findings apply to central and western regions of China or other countries requires further investigation.
4. The empirical scope of this study is correlational rather than causal. The methods used, social network analysis, coupled coordination models, and geographic detectors, are aimed at identifying associations and interaction patterns, not establishing causal relationships. Changes during the study period cannot be attributed solely to the pandemic, as they may also reflect simultaneous policy interventions and technological trends. Future research could use panel data econometric methods to strengthen causal inference.

## Data Availability

The raw data supporting the conclusions of this article will be made available by the authors, without undue reservation.

## Footnotes

1
http://finance.china.com.cn/news/20250301/6217827.shtml

2
http://www.jl.gov.cn/szfzt/tzcj/zdxm/szjj/202503/t20250320_3418400.html

3
http://www.bmronline.com.cn/index.php?m=content&c=index&a=show&catid=154&id=5123

4
https://ieknet.iek.org.tw/ieknews/news_open.aspx?actiontype=ieknews&indu_idno=3&nsl_id=79e89e42115840adbd1c4cacbd7f3fab

5
http://finance.people.com.cn/n1/2025/1029/c1004-40591680.html
